# Editorial: Artificial intelligence (AI) ethics in business

**DOI:** 10.3389/fpsyg.2023.1258721

**Published:** 2023-09-12

**Authors:** Alejo Sison, Ignacio Ferrero, Pablo García Ruiz, Tae Wan Kim

**Affiliations:** ^1^School of Economics and Business, University of Navarra, Pamplona, Spain; ^2^Department of Psychology and Sociology, University of Zaragoza, Zaragoza, Spain; ^3^Tepper School of Business, Carnegie Mellon University, Pittsburgh, PA, United States

**Keywords:** artificial intelligence, AI governance, artificial intelligence ethics, artificial intelligence in business, algorithmic decision-making

This Research Topic on Artificial Intelligence in Business delves into the multifaceted ethical dimensions of AI governance, exploring a variety of challenges and opportunities.

Artificial intelligence (AI) has become increasingly prevalent in businesses, revolutionizing how decisions are made and impacting various sectors such as e-commerce, transportation, and healthcare. Companies like YouTube, Amazon, Google, and Facebook leverage AI to personalize user experiences, while platforms like Uber and Lyft use it to match passengers with drivers and determine pricing. Similarly, Tesla's Advanced driver-assistance systems contribute to safer transport. These applications employ data-trained algorithms (“machine learning”) with minimal human intervention.

However, this growing reliance on AI raises significant ethical concerns and demands careful attention from managers and researchers alike. Several high-profile incidents have underscored the ethical challenges associated with AI adoption in business. Amazon's AI-driven recruitment tool demonstrated bias against women. Microsoft's chatbot, Tay, had to be discontinued due to racist and misogynistic remarks. And Tesla's autonomous systems have been involved in fatal accidents, leading to calls for greater public scrutiny.

Through this selection of papers, we uncover the ethical implications of AI in business and shed light on responsible governance practices to address them.

Daza and Ilozumba's paper conducts a comprehensive survey of business literature to identify the most influential journals, articles, and authors in AI ethics. It identifies the main ethical concerns and organizes them into five topic clusters: foundational issues; transparency, privacy, and trust; bias, preferences, and justice; jobs, employment and automation; and lastly, social media, participation and democracy.

Among foundational ethical issues, autonomy in decision-making is one of the most challenging. Moral agency attribution has traditionally been reserved for humans possessing rationality and freedom. However, AI systems are designed precisely to make decisions autonomously. With self-driving vehicles, robotic caregivers, autonomous weapons, and so forth concerns about loss of control loom large. This requires investigations into the extent to which moral attribution applies to AI, and whether recognizing AI agency necessitates a reframing of ethical frameworks. Exploring parallels between AI agency and corporate agency from legal, moral, and psychological perspectives could shed light on this complex subject.

Bertoncini and Serafim argue that as AI becomes more integral to our lives, AI ethics should no longer be viewed as peripheral but rather as an intrinsic requirement. They suggest examining moral agency and AI in three critical points, namely: autonomy, right of explanation, and value alignment. Thus, they pave the way for a deeper reflection on human and artificial intelligence interaction. In the same vein, De Cremer and Narayanan advocate retaining human responsibility in decision-making despite AI advancements. AI can play a crucial role in enhancing ethical decision-making by serving as a mirror that reflects our biases and flaws, ultimately helping humans gain a better understanding of ethical choices and behaviors.

Two other salient ethical topics for AI use in business refer to transparency and bias. On the one hand, AI algorithms often operate as “black boxes”, making it challenging to understand how decisions are made and whether or how to hold them accountable for potential biases or errors. On the other hand, AI systems reflect and amplify societal biases present in the data on which they are trained.

Three papers address the challenges of mitigating bias and promoting fairness in algorithmic decision-making. Leben focuses on the importance of explanations in AI decision-making and explores the role of counterfactuals in fair deliberations. Based on the perceptions of laypeople, Claudy et al. investigate how the acceptance of AI as a replacement for human decision-makers is influenced by perceived impartiality. While people attribute greater impartiality to AI, their preference for human decision-makers can change when human biases are made salient. Piccininni underscores the lack of consensus on an operational definition of fairness. By means of a case study on the reputational-ranking algorithm used by a food delivery platform, he examines the applicability and intuitiveness of causal models in evaluating fairness, highlighting the alignment of causal-based fairness definitions with human conceptions of fairness.

The widespread deployment of AI in business has provoked upheavals in the labor market. Concerns vary from the loss of jobs due to automation, through the proliferation of precarious jobs in the on-demand (or “gig”) economy, to the transformation of work and work relations. Focusing on personnel selection, Kupfer et al. rehearse possible strategies to reduce automation bias in AI-based decision support systems. Providing information about system errors and decision-makers' responsibility, along with the appropriate level of data aggregation can enhance decision quality and mitigate automation bias. For their part, Redín et al. study the relationship between innovation, robots, and AI in the context of a changing labor market. Innovation is inherent to human nature alongside engagement with traditions. Thus, the capacity to innovate is distinctively human, with machines playing the limited role of tools.

Together, these papers offer a comprehensive exploration of ethical issues in AI governance in business and hope to contribute to the development of practices that prioritize individual human and societal wellbeing.

## Author contributions

AS: Writing—original draft. IF: Writing—original draft. PG: Writing—review and editing. TK: Writing—review amd editing.

